# Efficacy of artesunate–amodiaquine, dihydroartemisinin–piperaquine and artemether–lumefantrine for the treatment of uncomplicated *Plasmodium falciparum* malaria in Maradi, Niger

**DOI:** 10.1186/s12936-018-2200-1

**Published:** 2018-01-25

**Authors:** Francesco Grandesso, Ousmane Guindo, Lynda Woi Messe, Rockyath Makarimi, Aliou Traore, Souleymane Dama, Ibrahim Maman Laminou, Jean Rigal, Martin de Smet, Odile Ouwe Missi Oukem-Boyer, Ogobara K. Doumbo, Abdoulaye Djimdé, Jean-François Etard

**Affiliations:** 1 Epicentre, 8 rue Saint-Sabin, 75011 Paris, France; 2 Epicentre Research Centre, Maradi, Niger; 3 Malaria Research and Training Centre, Department of Epidemiology of Parasitic Diseases, Faculty of Pharmacy, University of Science, Techniques and Technologies of Bamako, P.O. Box: 1805 Point G, Bamako, Mali; 4 Parasitology Unit, Centre de Recherche Médicale et Sanitaire, Niamey, Niger; 5 Médecins Sans Frontières, 8 rue Saint-Sabin, 75011 Paris, France; 6 Médecins Sans Frontières, rue de l’Arbre Bénit 46, 1050 Brussels, Belgium; 7 IRD UMI 233, INSERM U1175, Unité TransVIHMI, Université de Montpellier, 34000 Montpellier, France

**Keywords:** Malaria, Efficacy, Antimalarial, Artemisinin, Resistance, Parasite clearance, Niger

## Abstract

**Background:**

Malaria endemic countries need to assess efficacy of anti-malarial treatments on a regular basis. Moreover, resistance to artemisinin that is established across mainland South-East Asia represents today a major threat to global health. Monitoring the efficacy of artemisinin-based combination therapies is of paramount importance to detect as early as possible the emergence of resistance in African countries that toll the highest burden of malaria morbidity and mortality.

**Methods:**

A WHO standard protocol was used to assess efficacy of the combinations artesunate–amodiaquine (AS–AQ Winthrop^®^), dihydroartemisinin–piperaquine (DHA–PPQ, Eurartesim^®^) and artemether–lumefantrine (AM–LM, Coartem^®^) taken under supervision and respecting pharmaceutical recommendations. The study enrolled for each treatment arm 212 children aged 6–59 months living in Maradi (Niger) and suffering with uncomplicated falciparum malaria. The Kaplan–Meier 42-day PCR-adjusted cure rate was the primary outcome. A standardized parasite clearance estimator was used to assess delayed parasite clearance as surrogate maker of suspected artemisinin resistance.

**Results:**

No early treatment failures were found in any of the study treatment arms. The day-42 PCR-adjusted cure rate estimates were 99.5, 98.4 and 99.0% in the AS–AQ, DHA–PPQ and AM–LM arms, respectively. The reinfection rate (expressed also as Kaplan–Meier estimates) was higher in the AM–LM arm (32.4%) than in the AS–AQ (13.8%) and the DHA–PPQ arm (24.9%). The parasite clearance rate constant was 0.27, 0.26 and 0.25 per hour for AS–AQ, DHA–PPQ and AM–LM, respectively.

**Conclusions:**

All the three treatments evaluated largely meet WHO criteria (at least 95% efficacy). AS–AQ and AL–LM may continue to be used and DHA–PPQ may be also recommended as first-line treatment for uncomplicated falciparum malaria in Maradi. The parasite clearance rate were consistent with reference values indicating no suspected artemisinin resistance. Nevertheless, the monitoring of anti-malarial drug efficacy should continue.

*Trial registration details* Registry number at ClinicalTrial.gov: NCT01755559

## Background

Malaria is a leading cause of morbidity and mortality in Niger, with 5.2 million cases and 10 thousands deaths being estimated in 2015 [1]. The first-line treatment is since 2005 artemether–lumefantrine (AM–LM), one of the most widely used artemisinin-based combination therapy (ACT). Anti-malarial treatment policy was revised in 2008, introducing artesunate–amodiaquine (AS–AQ) as alternative first-line treatment, along with the artemether–lumefantrine paediatric suspension [2]. Two studies carried out with the support of the National Malaria Control Programme demonstrated 92% efficacy for AM–LM in 2005–2006 [3], and 94.8 and 97.1% for AM–LM and AS–AQ, respectively in 2011 [4].

Resistance to artemisinin emerged in Thailand–Cambodia border in the early 2000 [5–7]. Resistance is established across mainland South-East Asia, and, although there is no documented evidence of spread to the African continent [8], it represents today a considerable threat to global health. Delayed parasite clearance is an early indicator of the emergence of resistance to artemisinin [6, 9, 10]. Artemisinin resistance in South-East Asia was associated with specific mutations in the “propeller region” of the *Plasmodium falciparum* kelch protein gene (*Pfk13*) [8, 11]. A recent study in the Niger capital, Niamey, highlighted the presence of mutations in *Pfk13*, although none of them were among the mutations associated with artemisinin resistance in South-East Asia [12].

In the effort to monitor the efficacy of anti-malarial treatments in use, a three-arm efficacy study was carried out in Maradi, in the south of Niger. AM–LM and AS–AQ were evaluated, as well as dihydroartemisinin–piperaquine (DHA–PPQ). This latter treatment has proven to be highly efficacious in previous studies [13–15] and received favourable opinion of the European Medicines Agency in June 2011 [16], just before this study was started. This treatment was therefore a potential good alternative first-line treatment, if the recommended ACT showed an efficacy below the threshold recommended by the World Health Organization (WHO) [17].

## Methods

### Study overview

This was an in vivo study in children 6–59 months with a *P. falciparum* mono-infection confirmed with microscopy. Eligibility for inclusion and clinical and parasitological evaluations were according to WHO standardized protocol for monitoring anti-malarial drug efficacy [18]. In addition, parasite clearance was assessed using a standardized method [10] to monitor the possible emergence of resistance to artemisinin. The study took place at a health facility of Epicentre research centre located in the compound of the Integrated Health Centre (CSI) of Andoumé in Maradi. The CSIs of Andoumé and Dix-sept Portes, both located in the town of Maradi, provided eligible patients among CSI attendants.

### Sample size

With an expected efficacy of 95%, an accuracy of 3%, the sample size was estimated to be 184 children per arm. This sample size allowed to test the hypothesis that the expected efficacy of 95% was not statistically equal or inferior to 90%, with an alpha error of 0.05 and a power of 80%. Sample size was increased of 20% to take into account the occurrence of re-infections, indeterminate PCR results and loss to follow-up. The final sample size was 221 children per arm, and therefore 663 children in total.

### Enrolment and study treatments

Children meeting inclusion criteria were enrolled and treated on-site with a 3-day regimen of either artesunate–amodiaquine (AS–AQ Winthrop^®^ Sanofi Aventis), dihydroartemisinin–piperaquine (DHA–PPQ, Eurartesim^®^ Sigma-Tau) or artemether–lumefantrine (AM–LM, Coartem^®^ Novartis). All treatments were given under supervision of a nurse and respecting pharmaceutical recommendations (AS–AQ administered once a day with no special recommendations; DHA–PPQ administered once a day at no less than 3 h after the last food intake and no food within 3 h after each dose; AM–LM administered with a glass of milk, the second dose at 7–8 h after the first dose and subsequent doses every 12 h). The allocation of patients to one of the three treatments was randomized. This procedure allowed carrying out inclusions in parallel, and provided a better representativeness of the study population throughout the study period for each of the study treatment.

After admission (day 0), children were followed-up at pre-determined intervals (day 2, 3, 7, 14, 21, 28, 35 and 42) and at any time the care giver judged the child did not feel well. At each visit, both thick and thin blood smears were taken from enrolled patients. Microscopic examination was done under 100× oil immersion magnification. A physician examined the child’s clinical condition during the entire follow-up period.

### Study endpoints

Patients were classified as (1) early treatment failure (ETF), (2) late clinical failure (LCF), (3) late parasitological failure (LPF), or (4) adequate clinical and parasitological response (ACPR), as per WHO guidelines [18]. Children were withdrawn from the study at any time during the follow-up period if they met one or more of the following criteria: (1) failure to take any study treatment dose; (2) development of an allergic reaction to the study treatment or a side effect severe enough to require an alternative treatment; (3) detection of a non-falciparum mixed or mono-infection; (4) occurrence of a severe infectious disease; (5) self-medication with any anti-malarial drug during follow-up; or (6) consent withdrawal by the parent or the guardian. Children were classified as lost to follow-up if they did not attend the day 42 visit (1 day of delay was accepted).

### PCR

Samples for PCR analysis were collected on FTA cards, air-dried and stored in separated sealed bags in dry and dark conditions at room temperature. Samples were analysed at the Malaria Research and Training Centre in Bamako (Mali) using a previously described method [19].

Paired samples (pre-treatment and recurrent parasites) were compared and possible outcomes were: (1) recrudescence if similar alleles were found in the pre- and post-treatment samples for all the markers, (2) re-infection, if the alleles of the pre- and post-treatment samples were distinct; (3) mixed recrudescence and re-infection, if similar alleles were found in the pre- and post-treatment samples for all the markers as mentioned above, but with additional distinct alleles identified; (4) indeterminate, if at least one marker in either the pre- or the post-treatment sample did not allow a definitive conclusion; or (5) no DNA isolated, if one or both the pre- and post-treatment samples could not be amplified [19].

### Rescue treatment

Children who experienced therapeutic failure received a rescue treatment consisting of oral quinine as monotherapy at the dose of 10 mg/kg every 8 h for 7 days. In cases of severe malaria, the rescue treatment was quinine administered intravenously (20 mg/kg in the first 4 h, followed by 10 mg/kg during the following 8 h, then 10 mg/kg every 8 h for 7 days). With the administration of the rescue treatment, the normal study follow-up was terminated. However, patients who received rescue treatment were invited to return to the research centre at least once, to allow the research team to verify the efficacy of the treatment.

### Efficacy outcome analysis

Two analysis methods were used to estimate the therapeutic efficacy.

The Kaplan–Meier survival analysis was used to calculate PCR-adjusted estimates at day 28 and day 42 [18, 20]. Patients who discontinued treatment, were lost to follow-up or had a re-infection were censored starting from the last day the patient was seen. Patients with a recurrent parasitaemia, but without PCR result or with indeterminate result, were censored at the last visit with a negative blood smear. Excluded from this analysis were patients that were erroneously included in the study (i.e. not responding to all inclusion and exclusion criteria).

The second analysis used the per-protocol method and was carried out to allow a comparison with previous studies. In this method, the efficacy was estimated as proportion of ACPR and was based only on the patients for which a therapeutic endpoint (ETF, LCF, LPF and ACPR) was reached. All other patients, including reinfections, were excluded from this latter analysis.

Percentages and rates were expressed with 95% confidence intervals. Quantitative variables were described by mean and standard deviation if normally distributed, and by median and interquartile 25 and 75 percentiles range (IQR), if not normally distributed. Comparison of continuous numeric variables was tested with the one-way analysis of variance (ANOVA) if normally distributed, and with Kruskal–Wallis test if not normally distributed.

### Parasite clearance

A blood smear was collected every 8 h (with a tolerance window of 2 h) starting from the first treatment dose intake. Sampling for parasite clearance was stopped when two consecutive blood smears were negative. As ETF endpoint requires a blood smear sample at day 3, this latter was always performed even if most of patient’s blood smears performed for the parasite clearance were negative by day 1 or day 2 [6]. The clearance rate constant and the slope half-life were used to measure the parasite clearance as proposed by Flegg and colleagues [10]. The statistical models used to estimate the parasite clearance measures and lag phase duration were fitted using the Parasite Clearance Estimator developed by the World Wide Antimalarial Resistance Network (WWARN) [21].

### Safety

The safety and tolerability of the three treatments were previously assessed and treatments were recommended for routine clinical use [13, 14, 22, 23]. However, clinicians were asked to describe any adverse events that occurred during the patient’s follow-up (nature, severity, probable association with the treatment studied and evolution). Similarly, any serious adverse event resulting in death, putting the patient’s life in danger or resulting in disability, significant impairment, or leading to hospitalization were immediately notified to the principal investigator who referred the case to an external reviewer of serious adverse events. Definition used were as proposed by the International Conference of Harmonization [24, 25].

### Ethical considerations

Written informed consent was obtained from the parent or the guardian of each child enrolled.

The MSF Ethical Review Board and the National Ethics Committee of the Ministry of Health of Niger approved the study protocol. The study was registered at ClinicalTrials.gov (NCT01755559).

## Results

### Baseline characteristics

Between 17 June 2013 and 22 September 2014, of 1141 children aged 6–59 months who attended the study clinic (918 from Andoumé health centre and 223 from Dix-sept Portes health centre), 663 children (221 in each study arm) satisfied the inclusion criteria, and were enrolled in the study.

**Fig. 1:**
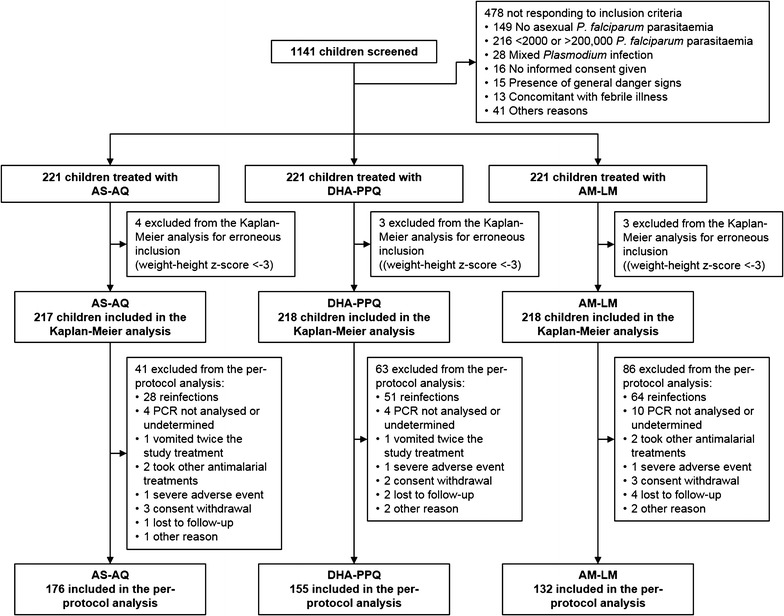
Study flowchart

Of the 221 children included in each treatment arm, 4, 3 and 3 children were excluded from the Kaplan–Meier analysis in the AS–AQ, DHA–PPQ and AM–LM arms respectively, while, in the same order, 41, 63 and 86 children were excluded from the per-protocol analysis. The study flowchart, with the reasons for excluding from the analyses, is presented in Fig. 1.

The median age was 29, 29 and 30 months and the male/female ratio was 1.1, 1.2 and 1.1 for AS–AQ, DHA–PPQ and AL–LM respectively. No significant differences on baseline characteristics were found between treatment arms (Table 1).

**Table 1:** Baseline characteristics of patients admitted to the study

	AS–AQ (N = 217)		DHA–PPQ (N = 218)		AM–LM (N = 218)		*p*
Age (months)—median [IQR]	29	[18–41]	29	[18–39]	30	[21–39]	0.898
Sex ratio (M/F)	1.1	(115/102)	1.2	(118/100)	1.1	(112/106)	0.846
Weight—median [IQR]	10.2	[8.5–12.5]	10.5	[9.1–12.1]	10.5	[8.7–12.3]	0.975
Weight Z-score—mean (SD)	− 0.85	(0.92)	− 0.85	(0.87)	− 0.97	(0.89)	0.324
Axillary temperature °C—mean (SD)	39.0	(1.1)	39.0	(1.1)	38.9	(1.1)	0.666
Temperature ≥ 37.5 °C—n (%)	194	(89.4)	197	(90.4)	197	(90.4)	0.927
Temperature ≥ 38.5 °C—n (%)	152	(70.1)	158	(72.5)	150	(68.8)	0.694
Parasite density/µL—median [IQR]	34,915	[9822–77,185]	41,042	[14,118–85,926]	46,506	[13,776–98,311]	0.111
Presence of gametocytes—n (%)	11	(5.1)	5	(2.3)	9	(4.2)	0.307
Haemoglobin (g/dL)—mean (SD)	9.5	(1.6)	9.7	(1.7)	9.7	(1.7)	0.286
Haemoglobin < 8 g/dL—n (%)	45	(20.7)	35	(16.1)	40	(18.4)	0.452

*IQR* Interquartile range, *SD* standard deviation

### Primary and secondary efficacy outcomes

In total 29, 57 and 78 children had a recurrent malaria infection in the AS–AQ, DHA–PPQ and AM–LM arms, respectively, during the 42 days follow-up. Following PCR analysis, 6 of them, 1, 3 and 2 in the AS–AQ, DHA–PPQ and AM–LM arms, respectively, were confirmed as recrudescence, either LCF or LPF. For 11 children with a recurrent malaria infection the PCR result was undetermined. No ETF were recorded in any treatment arm. The earliest treatment failure occurred at day 18 in the AM–LM arm.

**Fig. 2:**
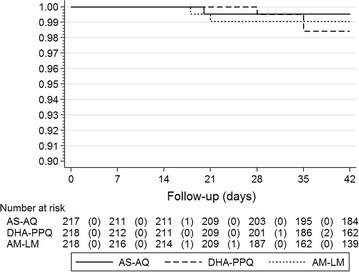
Kaplan–Meier PCR-corrected efficacy estimates or the three study treatments

The day-28 Kaplan–Meier efficacy estimates were 99.5% for AS–AQ and DHA–PPQ and 99.1% for AM–LM. The day-42 efficacy estimates, the primary efficacy outcome, were 99.5, 98.4 and 99.1% in the AS–AQ, DHA–PPQ and AM–LM arms respectively (Fig. 2).

The estimates as per-protocol analysis were similar of the Kaplan–Meier estimates (Table 2).

**Table 2:** PCR-corrected per-protocol efficacy estimates

	AS–AQ			DHA–PPQ			AM–LM		
	N	%	[95% CI]	N	%	[95% CI]	N	%	[95% CI]
ETF	0	0.0	–	0	0.0	–	0	0.0	–
LCF	1	0.6	[0.0–3.1]	1	0.6	[0.0–3.5]	1	0.8	[0.0–4.1]
LPF	0	0.0	–	2	1.3	[0.2–4.6]	1	0.8	[0.0–4.1]
ACPR	175	99.4	[96.9–100]	152	98.1	[94.4–99.6]	130	98.5	[94.6–99.8]
Total	176			155			132		

Fisher’s exact *p* = *0.778*

### Asexual parasite clearance

The median slope half-life (2.61, 2.68 and 2.77 h for AS–AQ, DHA–PPQ and AM–LM respectively) and the parasite clearance rate constant (0.27, 0.26 and 0.25 per hour for AS–AQ, DHA–PPQ and AM–LM respectively) were similar among the three treatments (*p* = 0.124), while the time to clearance 50 and 99% of parasitaemia was longer with AM–LM than with the other two treatments (*p* = 0.001) (Table 3). No patients presented asexual parasites on the blood smear microscopy 72 h after starting treatment.

**Table 3:** Parasite clearance indicators

	AS–AQ		DHA–PPQ		AM–LM		*p*
	Median	[IQR]	Median	[IQR]	Median	[IQR]	
Clearance rate constant (h)	0.27	[0.22–0.32]	0.26	[0.21–0.31]	0.25	[0.20–0.30]	0.124
Slope half-life (h)	2.61	[2.15–3.18]	2.68	[2.23–3.23]	2.77	[2.29–3.42]	0.124
Time to clearance 50% of parasitaemia (h)	9.10	[4.89–12.89]	8.79	[4.87–12.26]	10.10	[7.49–13.93]	0.001
Time to clearance 99% of parasitaemia (h)	24.41	[20.10–28.97]	23.79	[19.94–29.37]	26.31	[22.64–32.00]	0.001

### Gametocyte clearance

At admission gametocytes were detected in 5.1, 2.3 and 4.3% of children in the AS–AQ, DHA–PPQ and AM–LM arms, respectively. In the same order 2.4, 1.9 and 2.0% gametocytes were detected at day 3 (*p* = 0.930). Only one child in the AS–AQ arm was positive at day 14.

### Reinfections

**Fig. 3:**
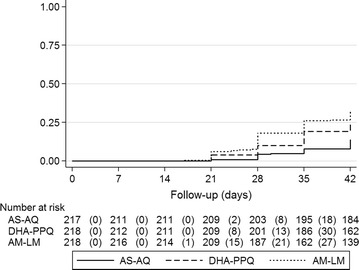
Reinfection rate expressed as a Kaplan–Meier probability of having a reinfection

During the 42-day follow-up, 28, 51 and 64 reinfections were reported in the AS–AQ, DHA–PPQ and AM–LM treatment arms, respectively. These events, expressed in terms of Kaplan–Meier failures, represent a risk of 13.8, 24.7 and 31.9% for AS–AQ, DHA–PPQ and AM–LM respectively (Fig. 3). The risk of reinfection was significantly higher with AM–LM (*p* < 0.001). In children taking AM–LM, the risk of reinfection was significantly higher for children aged 24–59 months (37.9%) compared to children aged 6–23 months (19.8%) (*p* = 0.007).

### Safety and severe adverse events

This analysis was performed by including all children in the study who took at least one dose of the treatment even if the treatment was not taken in full. Of the 221 children per treatment arm, the number of children with at least one adverse event during the follow-up period was 113 (60.2%), 132 (59.7%), and 140 (63.4%) for AS–AQ, DHA–PPQ and AM–LM, respectively.

The most common adverse events in decreasing order were fever, cough, rhinorrhoea, diarrhoea, conjunctivitis, pyoderma, vomiting and anorexia (Table 4). No child presented with myalgia, nausea or pruritus. One, two and one child respectively taking AS–AQ, DHA–PPQ and AM–LM presented splenomegaly (Table 4).

**Table 4:** Adverse events during the follow-up period

	AS–AQ (N = 221)		DHA–PPQ (N = 221)		AM–LM (N = 221)		*p*
	n	(%)	n	(%)	n	(%)	
Fever	90	(40.7)	94	(42.5)	94	(42.5)	0.906
Cough	31	(14.0)	36	(16.3)	22	(10.0)	0.141
Rhinorrhoea	20	(9.1)	27	(12.2)	17	(7.7)	0.255
Diarrhoea	19	(8.6)	14	(6.4)	15	(6.8)	0.624
Conjunctivitis	11	(5.0)	7	(3.2)	15	(6.8)	0.216
Pyoderma	12	(5.4)	6	(2.7)	6	(2.7)	0.211
Vomit	8	(3.6)	6	(2.7)	5	(2.3)	0.684
Anorexia	6	(2.7)	4	(1.8)	1	(0.5)	0.173
Asthenia	1	(0.5)	0	(0.0)	0	(0.0)	0.367
Abdominal pain	3	(1.4)	0	(0.0)	1	(0.5)	0.172
Arthralgia	1	(0.5)	0	(0.0)	0	(0.0)	0.367
Headache	1	(0.5)	0	(0.0)	0	(0.0)	0.367
Convulsions	1	(0.5)	0	(0.0)	0	(0.0)	0.367
Hepatomegaly	0	(0.0)	1	(0.5)	0	(0.0)	0.367
Splenomegaly	1	(0.5)	2	(0.9)	1	(0.5)	0.778
Other events	34	(15.4)	40	(18.1)	45	(20.4)	0.457

The number of events whose relationship to study treatment was classified as possible was 7, 8 and 4 for AS–AQ, DHQ–PPQ and AM–LM respectively. The number of events whose relationship to treatment was classified as probable was 1 for AS–AQ and DHA–PPQ and none for AM–LM. A total of 4 serious adverse events were reported; 1, 2 and 1 event with AS–AQ, DHA–PPQ and AM–LM, respectively.

In the AS–AQ arm, a child had decompensated anaemia (haemoglobin 5.3 g/dL) on the last day of treatment. At admission, parasitaemia was 2512 parasites/μL, haemoglobin level was 8.8 g/dL and the child had the spleen palpable under the costal rim. The relationship of the event to the study treatment was judged unlikely. The event was resolved in 5 days with a blood transfusion.

The first serious adverse event with DHA–PPQ was a child who experienced uncontrollable agitation requiring administration of sedatives on the second day of treatment. The relationship of the event with the study treatment was judged to be possible. The event was resolved.

The second serious adverse event with DHA–PPQ was a 14-month-old child who had a weight loss (weight 5.4 kg, height 72.0 cm) 19 days after the last treatment. At admission the weight was 6.6 kg. Weight loss was associated with a family event that resulted in a severe weaning of the child. The relationship of the event with the study treatment was judged unrelated. The child was treated at a nutritional centre and the event was resolved in 2 months (weight 7.4 kg, height 72.5 cm).

In the AM–LM arm, a child had had a respiratory infection requiring hospitalization and administration of antibiotics 20 days after the last treatment. The relationship of the event with the study treatment was judged unrelated. The event was resolved.

## Discussion

All the three anti-malarial treatments evaluated in this study showed to be very effective in treating uncomplicated falciparum malaria. The primary efficacy endpoint, the Kaplan–Meier efficiency estimate, but also other efficacy criteria, the per-protocol analysis and the parasite clearance indicators, yielded very reassuring results and were in line with similar studies in neighbouring countries [26–28].

No early therapeutic failure occurred and the estimated efficacies of all the three treatments were well above 95%, which is the WHO minimum threshold for recommending new anti-malarial treatments into the public health guidelines [17]. The three treatments can be recommended also for the population aged 5 years and older, for which, in addition to treatment, acquired immunity contributes to eliminate the parasite infection.

The three treatments showed that they are well tolerated, as only two children did not complete the treatment due to vomiting and only one serious adverse event, uncontrollable agitation requiring sedation in a child treated with DHA–PPQ, was thought to be associated with treatment. No adverse events were considered to have a highly probable relationship with the treatment. The number of other adverse events for which a relationship with treatment was considered to be possible or probable was quite low and their typology was similar to events reported in other studies [13, 14, 22, 29, 30].

The lengthening of parasite clearance time is retained as an early indicator of the emergence of artemisinin resistance [10]. The results of the parasite clearance indicators in this study were consistent with values indicating no resistance and were very far from values (half-life between 2.6 and 2.7 h in this study, versus 6.1 h in Pailin, Cambodia) where resistance to artemisinin has been demonstrated [6–8]. No resistance of artemisinin is confirmed by the absence of children who were parasitaemic on day 3 [31].

The only significant difference among the three study treatments between the three treatments was the number of reinfections that occurred during the follow-up period. While the high number of reinfections was expected, as most children were admitted to the study during the seasonal peak in the region, the number of reinfections after treatment with AM–LM (in almost one-third of children) was significantly higher than with the other two treatments. This might be accounted for the shorter half-life of lumefantrine [32] compared to amodiaquine [33] and piperaquine [34], that provided a shorter prophylactic effect as noted in other studies [35]. Nevertheless it might be an initial indication of the parasites reduce susceptibility to AM–LM, as suggested by Dama and colleagues [36].

## Conclusions

The results of this study indicate that the three treatments evaluated, AS–AQ, DHA–PPQ and AM–LM, largely meet WHO criteria. AS–AQ and AL–LM, the current first-line options, may continue to be used and DHA–PPQ may be recommended for the treatment of uncomplicated falciparum malaria in Maradi. Nevertheless, in the light of the emergence of artemisinin resistance in South-East Asia, their efficacy, as well as the molecular markers associated to artemisinin resistance, must continue to be monitored.
